# Comparative study: mapping treatment costs, best practices, and challenges across Africa

**DOI:** 10.3389/fpubh.2025.1690239

**Published:** 2025-12-24

**Authors:** Syed Ali Mehdi, Sultan Haider, Amro Kandil, Sebastian Ferrari-Stanford, Yonela Qwabe, Dahlia Hassan, Jude Shehadah, Jayati Vasavada, Mohd Mahmeen, Ankitesh Sinha, Samson Jarso, Nivisha Parag, Deogratias Mzurikwao, Bruno Sunguya

**Affiliations:** 1 Siemens Healthineers, Riyadh, Saudi Arabia; 2 Siemens Healthineers, Erlangen, Germany; 3 Siemens Healthineers, Cairo, Egypt; 4 Siemens Healthineers, Dubai, United Arab Emirates; 5 Siemens Healthineers, Johannesburg, South Africa; 6 Siemens Healthineers, Bengaluru, India; 7 Siemens Healthineers, Kemnath, Germany; 8 Johns Hopkins University, Baltimore, MD, United States; 9 Regent Business School, Durban, South Africa; 10 Muhimbili University of Health and Allied Sciences, Dar es Salaam, Tanzania

**Keywords:** Pan-Africa, treatment cost, infrastructure and access to care, comparative studies, liver cancer, lung cancer, coronary artery disease (CAD), stroke management

## Abstract

This paper presents the findings of the Siemens Healthineers SHIFT Innovation Pan-Africa Capacity Building Program, which aims to improve access and affordability of healthcare across Africa through knowledge sharing and the transfer of best practices. The study focuses on comparing the cost of treating liver cancer, lung cancer, coronary artery disease (CAD), and stroke in different African countries. These conditions represent a major and growing share of the non-communicable disease (NCD) burden across the African continent. They are also strategic focus areas for Siemens Healthineers due to their high clinical impact, complex diagnostic requirements, and the significant health system resources needed for effective management. These diseases serve as suitable proxies for assessing broader access challenges because they require timely detection, advanced imaging, laboratory diagnostics, specialized treatment pathways, and long-term follow-up care. Any gaps in diagnostic capacity, equipment availability, clinical workflow efficiency, workforce skills, or financing structures become immediately visible along these patient pathways. Comparing the differences with the best practices and challenges of each country, the programme aims to facilitate cross-cultural learning to bridge the gap between high- and low-value treatments. The study draws on detailed data on medical expenditure in several African countries, including public and private health services. Cost differences are analyzed taking into account medical procedures, drugs, diagnostic tests and hospital costs. In addition, the factors contributing to these cost variations, including healthcare delivery, resource availability, regulatory framework, and socio-economic factors, examined to develop standardized strategies to reduce medical costs and improve overall health outcomes. The results of this study will be a valuable resource for policy makers, healthcare providers and stakeholders to identify areas for improvement and take targeted actions. Ultimately, the aim is to improve access, affordability and quality of healthcare across Africa and ensure that life-saving treatments are readily available and affordable for all people on the continent.

## 1 Introduction

Access to high-quality healthcare is a fundamental right. In today's highly interconnected world, it should be available to everyone, everywhere, regardless of their geographical location or financial background. However, access to quality healthcare remains a major obstacle for many people in Africa. African healthcare systems are diverse and complex and vary greatly between countries and regions. While each country faces unique challenges, there are common themes across the continent such as limited resources, inadequate infrastructure, the digital divide and unequal access to healthcare. Each country is making its own efforts to improve its healthcare system. Nigeria, the most populous country in Africa and one of the fastest growing populations in the world, presents unique healthcare challenges. With a fertility rate of 5.5 live births per woman and an annual population growth rate of 3.2 per cent, the country is expected to reach a staggering population of 400 million by 2050 (1). Despite these remarkable demographic developments, the Nigerian healthcare system faces significant obstacles, mainly due to underdeveloped infrastructure, a lack of modern medical facilities and a shortage of healthcare professionals. The country loses at least 1.2 billion dollars annually through medical tourism (2).

According to the World Health Organization (WHO), Africa suffers from 25% of the global burden of disease, although Africa accounts for less than 1% of global health expenditure. Most Africans, especially the poor and those in the lower to middle income group, rely on underfunded public healthcare facilities, while a small minority have access to well-funded, high-quality private healthcare (3).

Lung cancer, liver cancer, stroke, and coronary artery disease (CAD) are major health problems in Africa and contribute to the burden of non-communicable diseases (NCDs) on the continent. While infectious diseases and other health problems remain prevalent, the incidence and mortality rates of these diseases are rising in many African countries. Risk factors for these diseases' include tobacco use, viral infections, aflatoxin poisoning, hypertension, diabetes, smoking, obesity, and physical inactivity (4). Tackling these diseases in Africa requires a multifaceted approach that includes smoking cessation, immunization, screening, and treatment of hepatitis infections, improving air quality, healthy lifestyle campaigns, and ensuring access to healthcare services for early detection, treatment and rehabilitation.

In South Africa, the healthcare system is relatively advanced compared to other African countries, with a mix of public and private healthcare providers. CAD is the most common cause of death in South Africa after HIV/AIDS. The country emphasizes primary prevention through public health campaigns to promote healthy lifestyles and manage risk factors. Specialized cardiac care units and emergency medical services ensure timely interventions, and access to diagnostic tests and treatment options. Research, training and collaboration with professional societies, human resources capacity and improved access to care are also ultimately helping to reduce the burden of CAD in South Africa (5).

Healthcare in Kenya continues to evolve, with a focus on implementing universal healthcare under the National Hospital Insurance Fund (NHIF), primary healthcare and maternal healthcare, non-communicable diseases and infectious diseases, to name a few. By investing in healthcare infrastructure, expanding health insurance coverage, strengthening of primary healthcare services, and introducing digital technologies to improve healthcare and data management, the Kenyan healthcare system is expected to experience an upswing in the coming years (6).

In contrast, the healthcare system in Egypt consists of a mixture of public and private providers. In recent decades, Egypt has achieved significant improvements in healthcare, which has led to an increase in average life expectancy (7). The country has a large number of medical facilities, and most Egyptian citizens live relatively close to a healthcare facility. However, the system faces challenges such as limited financial resources, insufficient quality of care and a lack of medical equipment and qualified staff in public healthcare facilities. As a result, private healthcare is favored by those who can afford it. The Egyptian government is actively promoting privatization while working toward achieving universal healthcare coverage by 2030 (8). Healthcare in Egypt continues to evolve, focussing on areas such as maternal and child health, lifestyle diseases, cancer treatment, stroke management, and geriatric care. By introducing new technologies, data-driven approaches and patient-centered care, the Egyptian healthcare sector aims to become a center for medical and wellness tourism, contribute to the country's GDP, and provide services in healthcare, pharmaceuticals, medical devices, medical education, and research.

The situation in these sample countries highlight the need for comparative studies and collaborative efforts to address healthcare challenges in Africa. Inspired by the overall situation on the continent, Siemens Healthineers (SHS), has actively collaborated with healthcare organizations in the region and developed a local innovation infrastructure (9). Siemens Healthineers SHIFT Innovation has conducted its 170th capacity building programme in a pan-African manner to map, look at, and validate trends in healthcare and hospitals relevant to access to care in Africa. This programme will serve as a platform for capacity-building and bring together key local healthcare institutions from countries such as Kenya, Nigeria, South Africa, Tanzania, and Egypt to work toward a common goal: access to care in Africa. By sharing experiences and best practices and collaborating on innovative solutions, these countries can collectively strive for better healthcare outcomes and ensure access to quality care for their populations.

Across the continent, the rising burden of non-communicable diseases has been widely documented, with regional analyses showing steady increases in cancer and cardiovascular mortality. Studies from the WHO African Region consistently highlight limited access to essential diagnostics, long travel distances to specialized centers, shortages of trained personnel, and heavy reliance on out-of-pocket payments for advanced care. While these reports provide important insights into individual countries and specific disease areas, much of the existing evidence remains fragmented. Most available studies examine single national contexts or focus on individual conditions, making it difficult to understand how different health systems compare or what drives variations in the cost and delivery of care.

There is a growing need for research that combines, pathway analysis and system-level context across multiple African settings. Understanding how treatment expenses differ between countries and how these differences relate to diagnostic capacity, service organization and financing arrangements. This essential for identifying shared challenges and opportunities for improvement. By examining treatment costs for four high-impact conditions across five countries, this study contributes a broader, comparative perspective that is often missing from the African NCD literature. The intention is to generate practical insights that can guide investment decisions, inform policy design and strengthen efforts to improve access and affordability.

African healthcare workers have limited access to training in targeted approaches to innovation management, and there is a need to build capacity at the local level. The aim of this comparative study is to analyze.

- Overall healthcare systems in African countries
- Transport issues faced by patients in terms of proximity to healthcare facilities and available transport methods
- Cost implications associated with different treatment options

Despite a very similar economic status, these countries have significant differences in their healthcare systems. By identifying best practices, trends and success factors, this study aims to help close the gap in healthcare provision and improve access to cost-effective, high-quality healthcare services both within and between these countries.

## 2 Material and methods

### 2.1 Programme design and participants

The Siemens Healthineers SHIFT Innovation Pan-Africa Capacity Building Programme, which took place from 23 March to 28 March 2023, was attended by 35 healthcare professionals, key opinion leaders (KOLs), from 14 African countries. The programme was designed to promote knowledge sharing and capacity building among trauma and therapy experts. The countries selected for the cost comparison study were Kenya, Nigeria, South Africa, Tanzania, and Egypt.

**Figure 1:**
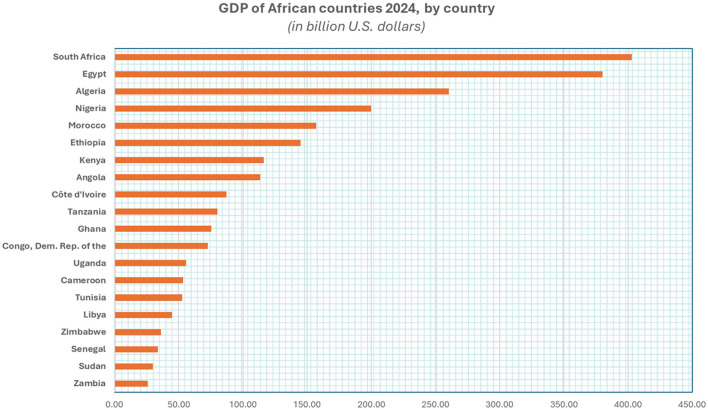
GDP African Countries 2004 by Statista.

These countries were chosen to ensure a representative mix across different economic tiers in Africa, enabling a meaningful and scalable comparison. As seen in the 2024 GDP data (Figure 1), South Africa and Egypt rank among the continent's highest economies, reflecting relatively advanced healthcare infrastructure and financing models. Nigeria, positioned in the mid-range, offers insights into managing healthcare in populous, resource-variable environments. Meanwhile, Kenya and Tanzania, with moderate to lower GDPs, bring forward the perspective of emerging health systems that face unique challenges in cost management and access to care.

This selection allows the study to capture a diverse range of contexts—from resource-rich to resource-constrained settings—providing a balanced view of treatment costs, best practices, and systemic healthcare challenges across Africa.

The programme aimed to foster impactful dialogue on healthcare innovation, equipping participants with strategies to address regional healthcare challenges effectively. Inspiring presentations by Key Thought Leaders (KOLs) from leading African organizations kicked off discussions on sustainable and accessible innovation programmes by addressing the five A's of access: Affordability, Availability, Appropriateness, Accommodation, and Acceptability.

- Availability: Whether the required services, equipment and specialists exist in the country
- Accessibility: Geographic reachability, travel distance and transport ease for patients
- Affordability: Direct and indirect patient costs, insurance coverage and catastrophic spending risk
- Acceptability: Cultural and patient-centered factors influencing willingness to seek care
- Accommodation: Health system ability to adjust to patient needs (appointment systems, waiting times)

These dimensions were scored through structured surveys and validated expert discussions.

### 2.2 Training and methodology

Prior to the start of the program, all 35 participants underwent standardized instruction in the Siemens Healthineers SHIFT Innovation framework, applied in more than 170 international innovation programs. The training covered ethical data handling, comparative cost-framework design, and statistical interpretation to ensure methodological consistency across countries.

### 2.3 Data collection

Cost and access data were collected for liver cancer, lung cancer, coronary artery disease (CAD), and stroke from five countries—Kenya, Nigeria, South Africa, Tanzania, and Egypt. Information was obtained through structured surveys, capacity-building workshops, and validated cost templates covering both public and private institutions. The data captured treatment costs, resource utilization, and access dimensions, ensuring comparability across diverse healthcare financing contexts.

### 2.4 Data validation

The data was validated by local experts and international experts from hospitals and universities, To strengthen reliability, a triangulated validation process was adopted involving (a) local expert review at Kenyatta University and University of Ibadan, (b) independent verification by Johns Hopkins University and Muhimbili University, and (c) The data was then further validated by the Siemens Healthineers SHIFT Innovation team through discussion with KOLs across Africa. This multilayered procedure enhanced data credibility despite the absence of centralized cost registries.

### 2.5 Statistical analysis

Comparative significance testing was conducted using the non-parametric Mann–Whitney *U* test to evaluate cross-country cost variations for major diseases. A two-tailed *p*-value of < 0.05 was considered statistically significant. This analysis was designed to identify associative trends between cost structures and systemic factors rather than infer direct causality and its qualitative comparative analysis. The results were subsequently visualized through comparative cost distribution charts and country-level mapping through value rating matrix. These detailed methodological clarifications enhance transparency and reproducibility, aligning the study with established standards for comparative health systems research.

### 2.6 Mapping and visualization

The results of the statistical analysis were used to create charts and maps that visually represent the differences in cost and access to healthcare in Kenya, Nigeria, South Africa, Tanzania and Egypt. Impact scoring followed a standardized Value Rating Matrix used in ITT programmes globally. Participants scored each proposed solution on:

- Impact (1–5 scale: low to transformative improvement)
- Effort (1–5 scale: minimal resources to high complexity)
- Maturity (1–5 scale: early concept to fully implementable)

Scores were averaged and discussed collectively to reduce evaluator bias; inter-rater variability was resolved through consensus discussion. This mapping helps to identify areas where costs and access to healthcare are particularly challenging.

### 2.7 Solution development and policy recommendations

During the process, each team representing 14 countries, identified specific challenges in their health systems related to the treatment of liver cancer, lung cancer, CAD and stroke and worked together to develop two alternative solutions for each of these challenges.

### 2.8 Programme facilitation

The programme was facilitated by the Siemens Healthineers SHIFT Innovation, which provided technical and logistical support to ensure that training, data collection, analysis and solution development ran smoothly. The organization commissioned collaboration with customers and fostered a spirit of mutual learning from each other's experiences.

## 3 Results

Based on the information collected through the Siemens Healthineers SHIFT Innovation Healthcare System Framework templates, a consolidated analysis was developed to evaluate treatment costs, availability of healthcare resources, and country-specific challenges and best practices related to access and affordability. Across all participating countries, the programme identified more than 300 healthcare trends, over 25 key opinion leader perspectives, insights from more than 15 stakeholder groups, eight recurring system pain points, more than 50 proposed solutions, and 14 focused solution clusters generated through group co-creation. These findings provide a structured overview of the systemic barriers faced at each stage of the care pathway and highlight potential interventions to strengthen access and affordability. The final consolidated dataset captured comparative insights across the five study countries on infrastructure readiness, diagnostic availability, specialist distribution, cost pressures and affordability challenges. These insights formed the basis for mapping cross-country differences in pathways for liver cancer, lung cancer, coronary artery disease and stroke.

### 3.1 Survey findings

The Pan-Africa Access to Care Dimension survey was divided into three categories to consolidate information and validate factors affecting access to care: (1) the availability and distribution of essential healthcare resources; (2) cost burdens related to specific disease treatments (liver cancer, CAD, stroke, and lung cancer); and (3) challenges and best practices.

Breakdown of Medical Expenditure—A detailed breakdown of medical expenditure was conducted for all four diseases across the five countries. Cost components were categorized as follows:

- Diagnostic services: imaging (CT, MRI, ultrasound), laboratory tests, pathology
- Therapeutic interventions: surgeries, chemotherapy, radiotherapy, interventional cardiology procedures
- Pharmaceuticals: oncologic medicines, cardiovascular drugs and supportive treatment
- Inpatient care: length of stay, ICU utilization, nursing care, and ward charges
- Outpatient and follow-up services: consultations, rehabilitation, and emergency visits

These categories were used to compare care pathways qualitatively across countries, identifying where the heaviest financial pressures typically arise.

### 3.2 Cross-country comparison (qualitative themes)

The qualitative comparative analysis revealed several consistent patterns across countries:

- Countries with limited diagnostic equipment faced higher indirect costs due to delayed diagnosis and longer pathways.
- Public–private cost differences were substantial, particularly in oncology care.
- Pharmaceutical costs emerged as a major affordability barrier in countries reliant on imported medications.
- Geographic disparities in specialist and facility distribution influenced both access and perceived cost burden.

## 4 Access to care factors and analysis

### 4.1 Cost burden

**Figure 2:**
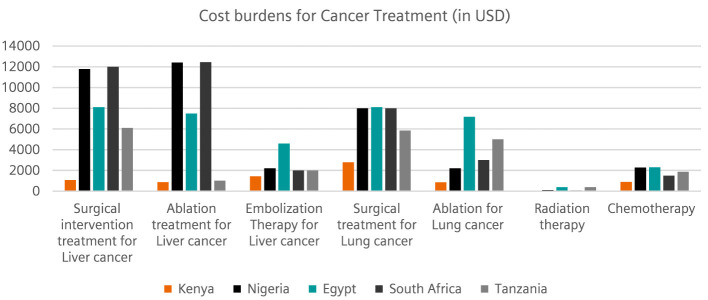
This illustration captures cost burdens *(USD per treatment)* related to cancer treatment with lung cancer and liver cancer in focus.

**Figure 3:**
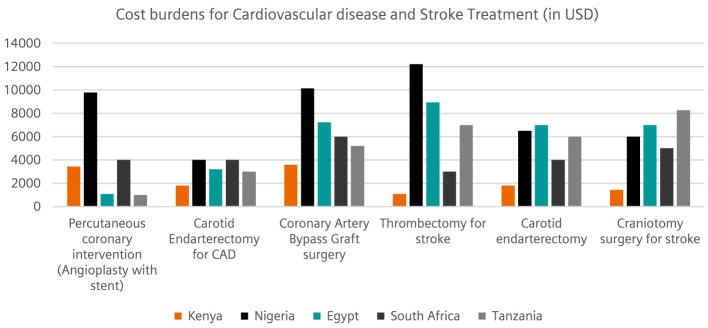
This illustration captures cost burdens *(USD per treatment)* related to cardiovascular diseases with CAD in focus and Stroke treatment. All cost figures are reported in United States Dollars (USD) for uniformity. Variations observed across countries reflect genuine differences in national health financing structures, exchange rates, and treatment coverage models rather than data inconsistency.

Based on the individual insights and observations, participants were also asked to identify the on different cost burdens for treating specific diseases (liver cancer, lung cancer, CAD and stroke). The inputs were categorized according to participants regions (Kenya, Nigeria, South Africa, Tanzania, and Egypt). The submissions indicated that Nigeria, South Africa, and Egypt had a higher cost burden for liver cancer treatment, while the cost of lung cancer treatment was comparatively lower in these regions. Kenya has a comparatively lower cost burden than other regions. In addition, Nigeria, Egypt, South Africa, and Tanzania had a higher cost burden for the treatment of lung cancer. The costs of radiotherapy and chemotherapy for cancer treatment were relatively comparable in all regions (Figure 2). The highest cost burdens for the treatment of cardiovascular diseases and strokes were also observed in Nigeria, Egypt, Tanzania, and South Africa. Here too, the cost burden for Kenya remains low (Figure 3).

### 4.2 Best practices

**Figure 4:**
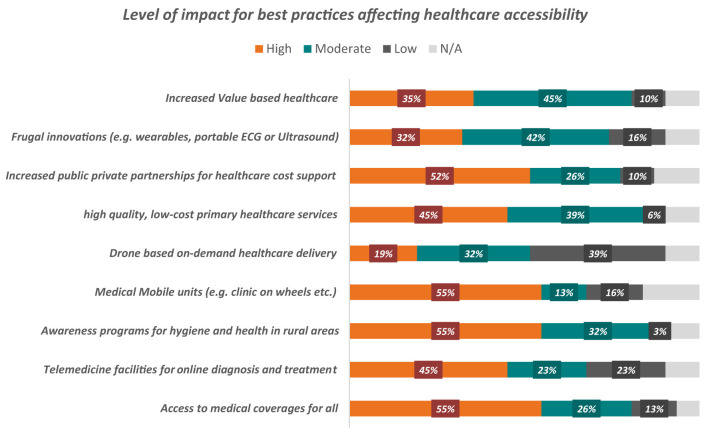
Outcome analysis for best practices in healthcare accessibility.

Best practices/possible solutions to achieve optimal accessibility to healthcare are illustrated in (Figure 4). Access to healthcare for all, hygiene and health awareness programme in rural areas and mobile medical units were rated with the highest impact (55%), followed by increased public-private partnerships to support healthcare costs (55%). 42% of participants said that frugal innovations had a moderate impact. Drones for on-demand healthcare delivery had have a low impact on healthcare accessibility.

Moreover, the responses collected on resource availability, cost burden, healthcare accessibility challenges and best practices during the workshop will be further utilized using the Innovation Methodology to co-ideate and co-implement the translational and innovative solutions to optimize healthcare accessibility. These insights reinforce the need for targeted and collaborative strategies to address healthcare accessibility challenges effectivelyBest practices/possible solutions to achieve optimal accessibility to healthcare are illustrated in (Figure 4). Access to healthcare for all, hygiene and health awareness programme in rural areas and mobile medical units were rated with the highest impact (55%), followed by increased public-private partnerships to support healthcare costs (55%). 42% of participants said that frugal innovations had a moderate impact. Drones for on-demand healthcare delivery had have a low impact on healthcare accessibility.

### 4.3 Challenges

**Figure 5:**
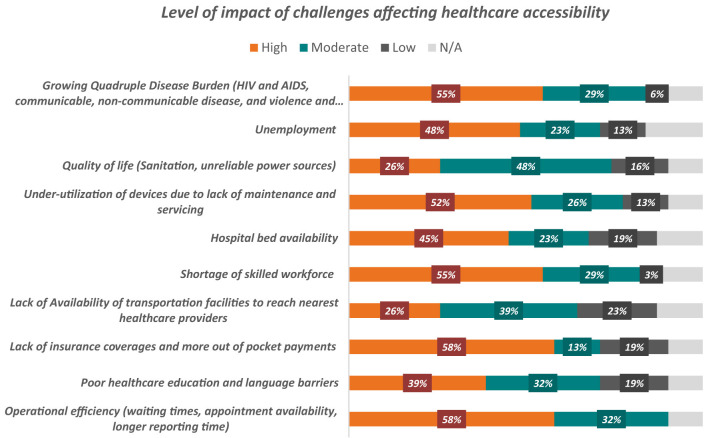
Outcome analysis for challenges in healthcare accessibility.

Healthcare accessibility aims to provide a positive patient experience. We mapped the main challenges during the workshop and asked participants to rate these according to their level of impact (Figure 5). Lack of insurance coverage and operational efficiency challenges had the greatest impact on accessibility according to 58% of participants, followed by the growing burden of disease and shortage of skilled workforce (55% each). Quality of life challenges had a relatively moderate impact on patient experience according to 48% of respondents.

## 5 Discussion

Kenya's low-cost health system for lung and liver cancer explains that it provides services to a large proportion of the population. Public health facilities provide affordable or subsidized healthcare, making it accessible to most Kenyans. The government invests in healthcare, staff, and essential medicines to control costs and ensure affordable healthcare. Kenya has introduced cost containment strategies in its healthcare system. The government regulates the prices of essential medicines and medical supplies, ensuring that they remain affordable to the public. In addition, regulations on medical care and pricing of services have been developed to help excessive healthcare costs. Kenya has a vibrant NGO sector linked to healthcare. Non-profit organizations, community health centers and mission clinics play an important role in providing affordable healthcare services to underserved communities. These organizations typically operate on low budgets and rely on grants or donations, which contributes to affordable healthcare in the country.

Kenya has a robust market for generic drugs, which helps to reduce the cost of drugs compared to generic alternatives. The availability of these affordable generics drugs helps to reduce the cost of healthcare in the country. Kenya has a critical shortage of well-trained healthcare professionals, including doctors, nurses and technicians. The high availability of healthcare professionals helps to meet the demand for healthcare services and leads to relatively low labor costs compared to countries without healthcare professionals. The availability of skilled healthcare professionals at affordable rates contributes to the overall affordability of healthcare in Kenya.

Kenya has endeavored to expand health insurance coverage for its citizens. The National Hospital Insurance Fund (NHIF) is a government health insurance scheme that covers a significant number of Kenyans (10). The NHIF helps individuals access cost-effective healthcare services by subsidizing medical expenses. Health insurance helps reduce the financial burden on individuals and lower the cost of healthcare.

**Figure 6:**
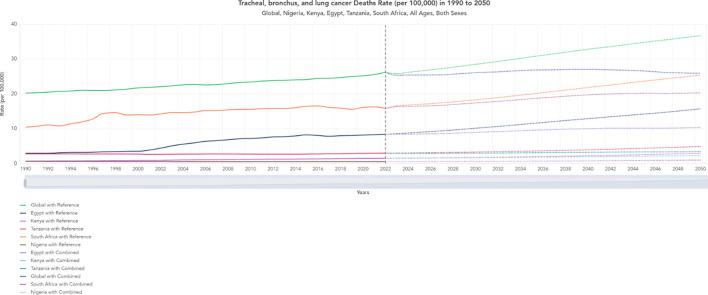
Historical trends and projections (1990–2050) for Tracheal, Bronchus, and Lung Cancer Death Rates (per 100,000).

**Figure 7:**
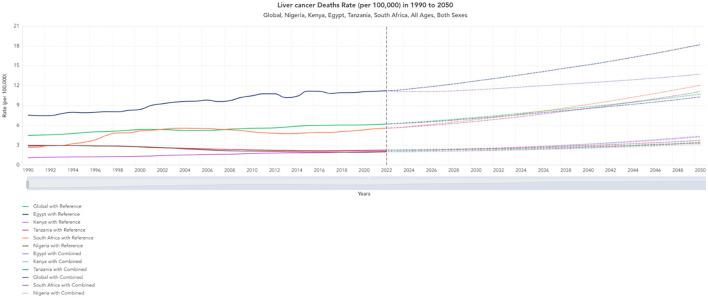
Historical trends and projections (1990–2050) for liver cancer death rates (per 100,000).

One of the factors contributing to the lower cardiovascular disease mortality rates in Kenya compared to the other countries featured in this study could possibly be due to the lower cost treatments that facilitate access to treatment options. The Figures 6, 7 shows the lower mortality rates in Kenya (Purple) for liver and lung cancer, which have been evolving since 1990 and are projected to follow a similar trend until 2050 (11). The annual proportion of deaths caused by cancer in the population is around 0.002% in Kenya, 0.02% in Egypt, 0.002% in Tanzania, 0.01% in South Africa, and 0.003% in Nigeria (12).

### 5.1 Similarities in medical expenditure between South Africa and Nigeria

- Statistical significance according to the Mann Whitney test comparing medical costs in South Africa and Nigeria was found to be insignificant in this study compared to other African countries. To investigate this further, they look at the best practices and challenges that may be associated with these similarities. Nigeria and South Africa are among the largest economies in Africa and have similar economic growth. In general, healthcare costs are linked to the economic status of the country, as rising income and higher living standards lead to higher healthcare costs. Nigeria and South Africa have a very well-developed healthcare system compared to many other African countries. It includes many hospitals, clinics, and healthcare facilities, and contributes significantly to the cost of healthcare. The availability of modern medical devices, advanced technologies, and skilled healthcare professionals increases the overall cost of healthcare.
- In both countries, both infectious and non-communicable diseases have a high disease burden. The prevalence of chronic diseases such as HIV/AIDS, malaria, tuberculosis, diabetes, cardiovascular diseases, etc. can lead to an increase in healthcare costs. The importance of prevention, treatment and management requires adequate funding.
- In Nigeria and South Africa, there is an extensive private healthcare sector that complements the public healthcare system. Private healthcare is often associated with higher costs due to factors such as better facilities, primary care, and better equipment. The availability and utilization of private healthcare contributes to the overall healthcare expenditure in these countries. Expenditure on drugs and medical supplies can have a significant impact on healthcare costs. Both Nigeria and South Africa rely on imported pharmaceuticals and medical supplies, which can be expensive due to factors such as import duties, transport costs and currency fluctuations etc. These costs also affect overall healthcare expenditure. These costs also have an impact on overall healthcare expenditure in the states.
- In Nigeria and South Africa, there is a mix of public and private healthcare. The public health system provides services to the majority of the population, which are usually subsidized or free. However, the quality and availability of public healthcare services can be limited, so some people seek treatment from private providers. Private healthcare services are often associated with high costs and contributing to overall healthcare costs. Additionally, healthcare policies and regulations can affect healthcare costs.

Factors such as pricing regulations, payment plans, and insurance coverage can affect the affordability and availability of healthcare. Nigeria and South Africa have different healthcare policies and regulations, but both countries face the challenge of balancing affordable care with quality care, resulting in similar healthcare costs.

### 5.2 Why CVD treatment is more expensive in Nigeria

In Nigeria, there are a few specialized hospitals or surgical centers that have the necessary facilities and equipment for CVD surgery. The increased demand and lack of competition resulting from this scarcity can drive up prices. CVD surgery requires the use of certain drugs, equipment, and materials. If these products have to be imported, costs may be higher due to factors such as customs duties, taxes, transport costs, currency fluctuations, etc. Local manufacturers or products that are in limited supply may also contribute to the reliance on expensive imported equipment.

Successful CVD surgery requires experienced cardiologists with extensive training and experience. The costs associated with recruiting and retaining well-trained surgeons can be significant and, potentially increase the price of CVD surgery. Stroke and post-operative care services, including intensive care unit (ICU) and specialized nursing care, add to the overall cost of CVD surgery, and if these services are limited or in high demand, this can increase the price.

Maintaining a well-managed operating theater requires a variety of operating costs, including electricity, water supply, maintenance and administrative costs. Inadequate infrastructure and the need for additional investment in these areas can drive up the price of CVD operations. Lack of government investment in adequate healthcare, including surgical facilities and infrastructure, can lead to higher costs. When public funding for healthcare is limited, private providers may charge more for reimbursement. The socioeconomic status of the population may also play a role in determining the cost of CVD surgery. If the population cannot afford affordable healthcare or health insurance, the costs are passed on to those undergoing surgery, driving up prices.

### 5.3 Tanzania and Egypt's unique efforts to make healthcare more affordable

In Egypt's healthcare system, public and private healthcare organizations coexist. The government plays a crucial role in controlling healthcare costs and ensuring accessibility. A significant portion of the Egyptian population receives subsidized healthcare services from public healthcare facilities, making access to necessary medical treatment affordable. To ensure that medicines and medical supplies remain affordable for the general public, the government controls their prices. In addition, Egypt has a developing non-profit sector that operates community health centers and clinics and provides accessible healthcare services to underserved communities. The availability of generic drugs in Egypt also contributes to the reduction of healthcare costs. Generic drugs are widely available in Egypt and can cost less than branded drugs. Thanks to this accessibility, the overall cost of healthcare is reduced.

Egypt has problems with a shortage of healthcare workers, like Kenya. Although there are more doctors, nurses and technicians than ever before, the demand for healthcare services is still enormous. The fact that there are so many qualified healthcare professionals available at relatively cheap labor costs contributes to the general affordability of healthcare in Egypt. Egypt has endeavored to facilitate access to health insurance for its residents. The Egyptian Health Insurance Organization (EHIO), a government health insurance programme, covers a significant portion of the population, improving access to healthcare and reducing the financial burden on individuals.

The emphasis on accessibility and affordability for the inhabitants of Tanzania is also reflected in its healthcare system. A large proportion of the population receives affordable or subsidized healthcare, mainly thanks to public health facilities. In order to reduce costs and provide accessible healthcare services, the government is investing in healthcare infrastructure, staff, and the necessary drugs. Like Kenya and Egypt, Tanzania relies on charity to close the gaps in healthcare provision in disadvantaged areas. In Tanzania, community health centers, missionary clinics and non-governmental organizations (NGOs) play an important role in providing accessible healthcare. These groups often have limited financial resources and rely on grants and donations to help underprivileged populations with the medical care they need. Although access to generic medicine is not as widespread in Tanzania as in some other countries, is still contributes to lower healthcare costs. Generic medicines offer more cost-effective treatment options for a variety of diseases.

Tanzania, like other African countries, is struggling with problems caused by a lack of qualified healthcare workers. But the government has endeavored to solve this problem by funding training initiatives and increasing the number of medical schools. Tanzania's National Health Insurance Fund (NHIF) hopes to improve access to affordable healthcare by providing residents with health insurance cover. The NHIF reduces the financial burden of medical bills and helps people access affordable healthcare services.

### 5.4 Transportation and its effects on access

In South Africa, Kenya, Nigeria, Tanzania, and Egypt, transport- problems are a major obstacle to patient's access to healthcare. The proximity of healthcare facilities and the availability of transport in all these countries have a significant impact on people's ability to receive timely and appropriate medical care. These challenges result in delayed healthcare, increased financial burden, barriers to accessing preventative services, overcrowded urban healthcare facilities, exacerbated health inequalities, increased risks to maternal and child health, and potentially life-threatening situations in emergencies.

While South Africa generally has a better developed healthcare infrastructure and with urban are areas well-connected by transport systems, problems persist in rural areas due to long distances involved. In Kenya, there are a variety of transport options in urban areas, while patients in rural areas are often dependent on motorbikes or walking. In Nigeria, the urban-rural divide is reflected in the accessibility of healthcare: there are more transport options in the cities, while motorbikes are common in rural areas. Transport can be difficult due to the poor condition of the road, congestion the cities and insecurities on the roads. Tanzania faces similar challenges, especially in the remote mountainous regions and on the islands, where boat transport is crucial. Egypt's urban areas have a variety of transport alternatives, although rural communities, particularly in desert regions, can still have problems with access. Despite the sophisticated infrastructure, access to healthcare can still be hindered by crowds and transport, especially in large cities such as Cairo. In some remote regions in the south of the country, access to transport services can also be limited.

All these countries are trying to improve transport infrastructure to improve healthcare access. Mobile health centers and telemedicine projects are often to reach neglected populations and make healthcare more accessible to all. In addition, the affordability and availability of private transport is crucial to improving the overall healthcare of the population.

### 5.5 Road transport comparative analysis

The total length of Kenyan road network is 160,886 km, of which 98,950 km are unclassified (13). This shows that there are few poorly maintained roads in rural areas of Kenya, which could make it difficult for residents to reach medical facilities. Access to healthcare may be affected by the condition of roads in both rural and urban areas as there is insufficient funding for road maintenance and reconstruction.

Road transport accounts for 90% of passenger and 75% of freight traffic in Tanzania (13). The trunk roads, on the other hand, are generally quite well-maintained, while the district and feeder roads, especially in the rural areas, can be in a poorer condition. However, this distinction in the classification of roads is crucial. Access to healthcare facilities in both rural and urban areas could be hampered by high accident rates due to poor road conditions.

Nigeria has a vast road network, but its condition continues to deteriorate due to lax maintenance and substandard construction. Access to medical treatment can be extremely difficult due to this deterioration, especially in remote areas during the rainy season. Transporting patients to medical facilities can be difficult due to potholes and uneven road conditions.

South Africa's extensive road network is partly maintained by regional or local road authorities. Access to healthcare services can be facilitated by a well-maintained national road network, especially in the major cities. As tolls can be expensive for patients and healthcare providers, the distinction between toll and non-toll motorways can have an impact on access to healthcare services.

The extensive road network in Egypt, which includes motorways and bridges, can make it easier for people to access healthcare facilities, especially in urban areas. However, the high volume of traffic in congested areas such as Cairo can still make it difficult to reach to medical facilities.

Both Egypt and South Africa have made large investments in building and modernizing their road networks, which could improve access to healthcare in urban and non-rural areas. Road maintenance and coordination problems in Tanzania could limit access to healthcare in both urban and rural areas. Poor maintenance of the extensive road network in Nigeria hinders access to healthcare, especially in rural areas. In Kenya, access to healthcare can be hampered by unclassified motorways, especially in rural areas with poor road maintenance upkeep.

To improve access to healthcare, especially in rural areas, all five countries could benefit from sharing best practices for managing road infrastructure, performing routine maintenance, and allocating funds. The emphasis on infrastructure development in Egypt and South Africa could serve as an example, while Tanzania and Nigeria should learn lessons from their difficulties and seek solutions to their road maintenance and funding problems. Kenya could benefit from better classification and maintenance techniques for roads.

### 5.6 Limitation

This study faced several methodological constraints inherent to multi-country health systems research. Data sources varied by country, with limited access to centralized cost repositories and reliance on validated but participant-supplied inputs. As this is a qualitative comparative analysis, variations in interpretation and contextual judgment are expected, since the study relies on expert insights rather than uniform quantitative cost datasets. Despite triangulated verification with academic and institutional partners, potential subjectivity remains a consideration. The qualitative nature of the study also means that comparisons emphasize themes, patterns and system-level differences rather than statistical generalization, which may influence how findings are interpreted across different contexts. Additionally, the analysis is cross-sectional and identifies associative relationships rather than causal effects. Future work should integrate longitudinal cost tracking and direct institutional audits to strengthen evidence precision and reproducibility. The absence of longitudinal or routinely collected administrative data further limits the ability to track cost evolution over time, which is typical of qualitative multi-country studies. Future work should integrate longitudinal cost tracking and direct institutional audits to strengthen evidence precision and reproducibility. Tt is also important to note that this paper is intended primarily as a reference and framework-level analysis rather than a definitive cost modeling exercise, and therefore serves as a foundational benchmarking tool to guide future, more data-intensive studies.

## 6 Conclusion

The Siemens Healthineers SHIFT Innovation Pan Africa Capacity Building Programme aims to strengthen healthcare access and affordability across Africa by promoting knowledge exchange, shared learning, and evidence-based best practices. This study compared treatment costs for liver cancer, lung cancer, coronary artery disease, and stroke across Kenya, Nigeria, South Africa, Tanzania, and Egypt, integrating insights from both public and private health systems. By mapping variations in diagnostic, treatment, pharmaceutical, and hospitalization costs, the analysis provides a clearer understanding of where financial barriers emerge within the patient pathway and how these barriers differ across countries.

Importantly, the comparative evidence generated by this study highlights specific levers through which access and affordability can be improved. Identifying countries with lower cost yet effective models of care enables others to adopt similar practices through pathway redesign, investment planning, and targeted capacity building. The evidence on cost drivers such as limited diagnostic availability, high out-of-pocket (Table 1) expenditure, or fragmented service delivery points directly to actionable system improvements, including decentralizing diagnostic services, optimizing referral pathways, improving equipment uptime, and adopting financing mechanisms that reduce the immediate financial burden on patients. By aligning cost insights with identified best practices, the programme enables stakeholders to prioritize high impact interventions that can make essential services more reachable, timely, and economically attainable.

**Table 1:** OOP health expenditure by country (2024).

**Country**	**OOP% of national health expenditure 2024**	**Analysis and recommendations**
Egypt	62.7%	The focus on universal health coverage shows a dedication to lowering out-of-pocket expenses and enhancing access to medical care. This action may reduce the financial burden on households and improve health outcomes (14–16)
Kenya	17.4%	Even if the percentage of out-of-pocket expenses is notable, the development of cutting-edge payment systems like CarePay shows efforts to advance payment methods. The report also highlights how vulnerable households are to rising healthcare costs, highlighting the need for further financial security (17, 18)
Tanzania	23.1%	The moderate amount of out-of-pocket spending in Tanzania shows that households there are under some financial strain, but it could also point to some ongoing initiatives to create insurance or healthcare (19)
Nigeria	80.4%	Nigeria's healthcare system mainly relies on out-of-pocket expenses, which suggests that there is little access to health insurance and may be a barrier to fair access to treatment. Insurance plans must be revised (20)
South Africa	6.45%	Nigeria's medical system the relatively low out-of-pocket costs in South Africa point to a more developed healthcare system with better insurance coverage or backing from the government (21)

Through collaborative engagement, countries discussed their progress, constraints, and health system innovations, enabling a shared understanding of what structures and policies most effectively reduce treatment costs and improve outcomes. This cross-country learning supports the development of standardized, scalable, and context-appropriate strategies that strengthen early detection, streamline care pathways, and enhance financial protection mechanisms.

Overall, the findings offer policymakers, health leaders, and system planners a practical evidence base to guide reforms aimed at reducing expenditure and improving accessibility. With many countries relying heavily on out-of-pocket payments, the study reinforces the need for sustained efforts in universal health coverage, innovative payment models, and strategic investments in diagnostic and treatment capacity. By learning from one another's successes and challenges, African countries can advance toward a future where high quality, life-saving care is both accessible and affordable for all populations across the continent.

## Data Availability

The datasets presented in this study can be found in online repositories. The names of the repository/repositories and accession number(s) can be found at: https://clients.ihme.services/sign-in?back=/data-explorer.
